# Shorter-duration escapes driven by *Drosophila* giant interneurons promote survival during predation

**DOI:** 10.1098/rspb.2024.1724

**Published:** 2025-05-28

**Authors:** Cynthia M. Chai, Carmen M. Morrow, Dhyey D. Parikh, Catherine R. Von Reyn, Anthony Leonardo, Gwyneth M. Card

**Affiliations:** ^1^ Janelia Research Campus, Howard Hughes Medical Institute, Ashburn, VA 20147, USA

**Keywords:** *Drosophila*, Odonata, escape behavior, giant fibre, predator–prey

## Abstract

Large axon-diameter descending neurons are metabolically costly but transmit information rapidly from sensory neurons in the brain to motor neurons in the nerve cord. They have thus endured as a common feature of escape circuits in many animal species where speed is paramount. Though often considered isolated command neurons triggering fast-reaction-time, all-or-none escape responses, giant neurons are one of multiple parallel pathways enabling selection between behavioural alternatives. Such degeneracy among escape circuits makes it unclear if and how giant neurons benefit prey fitness. Here we competed *Drosophila melanogaster* flies with genetically silenced giant fibres (GFs) against flies with functional GFs in an arena with wild-caught damselfly predators and found that GF silencing decreases prey survival. Kinematic analysis of damselfly attack trajectories shows that decreased prey survival results from predator capture of GF-silenced flies during some attack speeds and approach distances that would normally elicit successful escapes. In previous studies with a virtual looming stimulus, we proposed a model in which GFs enforce the selection of a short-duration take-off sequence as opposed to reducing reaction time. Our findings here demonstrate that, during real predation scenarios, the GFs indeed promote prey survival by influencing action selection as a means to increase escape probability.

## 1. Introduction

**Figure 1.**
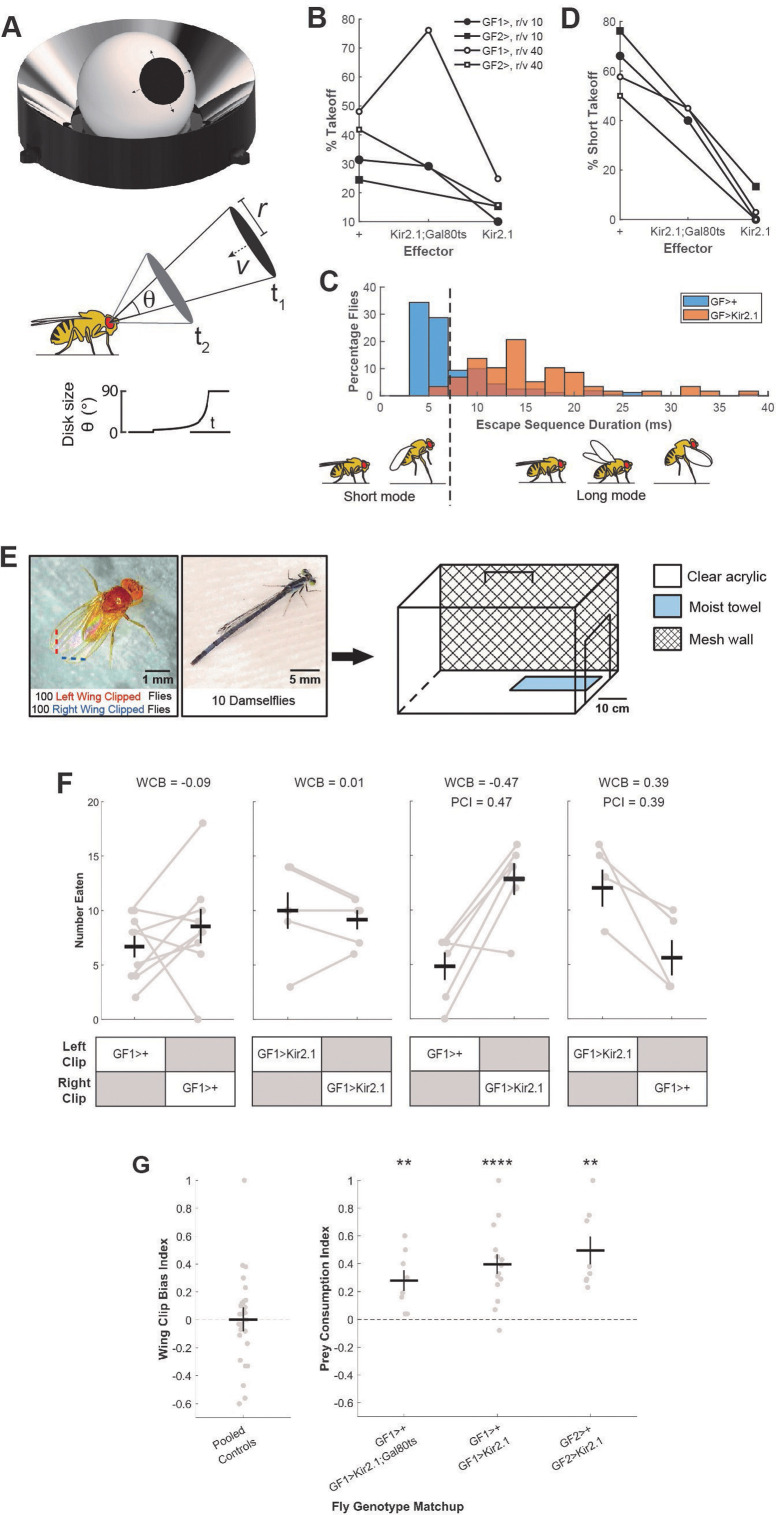
*Drosophila* GFs promote survival in an ecologically relevant predation regime. (A–D) We tested the effectiveness of genetically silencing the GFs in a virtual predation assay, the FlyPEZ (A), that displays visual looming stimuli to single flies (see Methods). (B) Percentage of flies that took off in the FlyPEZ assay in response to looming stimuli for two different GF-specific split-GAL4 driver line pairings (GF1, GF2) driving expression of the inwardly rectifying potassium channel Kir2.1 to hyperpolarize the GFs and prevent spiking. The Kir2.1; Gal80ts construct harbours fewer UAS copies, resulting in relatively lower Kir2.1 expression and a greater likelihood of GF spiking in GF>Kir2.1;Gal80ts flies relative to GF>Kir2.1 flies (see Methods). *r/v* = 10: GF1>+ (*n* = 188), GF1>Kir2.1; Gal80ts (*n* = 204), GF1>Kir2.1 (*n* = 139), GF2>+ (*n* = 86), GF2>Kir2.1 (*n* = 98) and *r/v* = 40: GF1>+ (*n* = 177), GF1>Kir2.1; Gal80ts (*n* = 222), GF1>Kir2.1 (*n* = 137), GF2>+ (*n* = 134), GF2>Kir2.1 (*n* = 89); *n* = number of videos analysed. (C) Histogram of escape sequence durations for flies with functional (GF>+) or silenced (GF>Kir2.1) GFs. The dashed vertical line indicates the intersection of short and long mode distributions. Data are pooled from experiments with both GF1 and GF2 drivers at both fast and slow looming speeds (*r/v* = 10, 40; see Methods). GF>+ (*n* = 160) and GF>Kir2.1 (*n* = 58). (D) Percentages of take-offs that were short mode. Fly genotypes and looming stimulus speeds as in (B). *r/v* = 10: GF1>+ (*n* = 59), GF1>Kir2.1; Gal80ts (*n* = 60), GF1>Kir2.1 (*n* = 14), GF2>+ (*n* = 21), GF2>Kir2.1 (*n* = 15) and *r/v* = 40: GF1>+ (*n* = 85), GF1>Kir2.1; Gal80ts (*n* = 169), GF1>Kir2.1 (*n* = 34), GF2>+ (*n* = 56), GF2>Kir2.1 (*n* = 14); *n* = number of videos analysed. (E) Prey survival competition assay. Each behaviour chamber was populated with 10 wild-caught damselflies and 200 flies: 100 with left distal wing tips excised (red dashed line) and 100 with right-wing tips excised (blue dashed line). Assays were run for 8 h. (F) Raw number of flies eaten for a subset of experiments with either the same (WCB) or different genotypes (PCI) in the left- and right-wing clipped groups. Grey dots connected by grey lines represent individual trials, black crosses represent mean ± s.e.m. WCB = wing clipping bias index, calculated as the difference in left and right-wing clipped flies eaten divided by the total number of flies (see Methods). PCI = prey consumption index, calculated as the difference in GF-silenced and GF driver-only control flies eaten divided by the total number of flies (see Methods). For both indices, a value of 0 indicates no bias in predation between the competed groups, and a value of ± 1 indicates that only members of one group were consumed. (G) Left—pooled WCB index scores for all genotypes (*n* = 30 trials total, see also electronic supplementary material, figure S1). Right—PCI scores for between genotype competition trials. Grey dots represent individual trials, black crosses represent mean ± s.e.m., *p*-values from one-sample *t*‐test. GF1>+vs. GF1>Kir2.1; Gal80ts (*n* = 8, **p = 0.0068), GF1>+vs. GF1>Kir2.1 (*n* = 15, ****p = 6.0338E−5), GF2>+vs. GF2>Kir2.1 (*n* = 8, **p = 0.0017); *n* = number of trials.

The large axonal diameter of giant descending interneurons enables rapid information transmission between sensory neurons in the brain and motor neurons via the ventral nerve cord [1]. Despite the high energetic costs of maintaining such a large cell [2–4], giant interneurons are a common feature of escape circuits in many animal species where a rapid response is paramount [1,5–9]. Although commonly regarded as command neurons that serve as the all-or-none decision-making control element that quickly triggers an escape response [10,11], giant neurons are just one of the multiple parallel pathways collectively forming a larger escape network [12–17]. In squid, for example, the interplay between giant and non-giant pathways enables behavioural flexibility during the escape response [17]. Crayfish can also generate tail flip escape responses via non-giant pathways in response to abrupt tactile threats [12,14]. Similarly, in flies, genetic silencing of the giant fibres (GFs) does not eliminate visually evoked escape take-offs, although without GF activity, flies only perform longer-duration take-off sequences that are less likely to launch them off the ground before a theoretical time-of-contact with a virtual stimulus (see also figure 1*a*,*c*) [18].

Depending on the approach speed of an artificial looming visual stimulus, the fruit fly *Drosophila melanogaster* selects between executing a fast escape take-off motor programme or a slower but more stable take-off sequence [18]. The switch to a short mode escape depends on GF activation, which can override alternate parallel descending pathways to truncate the escape sequence and propel the fly out of harm’s way once angular size and speed thresholds have been reached [18]. In the wild, perching flies are at risk of being caught by predators such as the carnivorous odonate, the damselfly [19,20]. Recordings of damselfly attack trajectories indicate that they can attack at speeds near the edge of the fly’s ability to respond [18]. However, the relationship between GF activity and fly survival when confronted with real predatory danger has not been directly established. Given the degeneracy present in fly escape circuits and the observation that the GF is not necessary for looming-evoked longer-duration take-offs [18], does the GF confer a survival advantage when a fly is challenged with a real predator?

## 2. Results

### (a) *Drosophila* giant fibres promote survival in an ecologically relevant predation regime

We leveraged the extensive *Drosophila* transgenic toolkit to achieve constitutive inhibition of only the two bilaterally paired GF interneurons in the fly’s nervous system [21–23]. Cell-type specificity was achieved using the split-GAL4 intersectional strategy, where only neurons that express both the DNA-binding domain (DBD) and activation domain (AD) components driven by different enhancers will reconstitute a functional GAL4 transcription factor [22]. To generate flies with silenced GFs (‘GF>Kir2.1’), we crossed transgenic strains harbouring GF-specific split-GAL4 driver constructs (either GF1 or GF2) with a strain bearing the GAL4-binding upstream activating sequence (UAS) that drives expression of the inwardly rectifying potassium channel Kir2.1, which hyperpolarizes the GFs and inhibits neuron spiking [21]. Control flies (‘GF>+’) with a similar genetic background but lacking Kir2.1 channel expression were generated by crossing GF-specific split-GAL4 driver strains with a wild-type strain (Dickinson Lab strain, DL). To confirm that flies expressing Kir2.1 in the GFs had compromised escape responses, we used an automated high-throughput behaviour system, the FlyPEZ [24], to present flies with a virtual looming stimulus that mimics a dark object approaching the fly at either a slow (*r/v* = 40) or fast (*r/v* = 10) constant velocity (figure 1A, see Methods). We recorded the time of take-off for each fly in response to the looming stimulus and calculated the likelihood that flies took off before a hypothetical time-of-contact with the oncoming virtual object. Fewer flies with Kir2.1-expressing GF neurons took off in response to looming compared to the split-GAL4 driver-only controls (figure 1B). For the flies that took off, we measured the duration of the take-off sequence (time from start of wing raising to time tarsi lost contact with the ground) to determine whether each take-off was short (< 7 ms) or long (> 7 ms) mode [18]. At both slow and fast looming speeds, GF>+ take-offs were biased towards the faster, short mode escape programme (figure 1C,D). However, short mode escapes were almost completely eliminated for GF>Kir2.1 flies (figure 1C,D). Moreover, flies expressing the Kir2.1 effector showed greater short mode escape reduction compared to the weaker Kir2.1; Gal80ts effector, which has a lower UAS copy number (figure 1D, see Methods). This confirms our previous observations that the GFs drive short mode take-offs, whereas visually mediated long mode escapes are controlled by alternative non-GF neural pathways [18].

To determine whether GF-mediated short-mode escapes confer a survival advantage under naturalistic predation conditions, we developed a novel prey survival competition assay. In the assay, flies of two different genotypes interacted freely with their natural predators, damselflies, within four custom-built chambers placed in an indoor arena simulating outdoor summer environmental conditions (figure 1E, electronic supplementary material, figure S1A). All damselflies used in this study were wild-caught each morning from two sampling sites along a pond within the Janelia Research Campus (electronic supplementary material, figure S1B). As GF-mediated escape performance does not rely on wing functionality [25], fly genotype was marked by excising a small portion of each fly’s wing tip on either the left or right side. Each trial lasted 8 h, beginning mid-morning and ending in the early evening. At the end of each trial, uneaten flies were anesthetized, sorted based on the wing-clipped side, and counted.

As a control, we first competed flies of the same genotype but with different wing sides clipped against each other. A negative wing clipping bias (WCB) index value indicates that more right-wing clipped flies were eaten compared to left-wing-clipped flies, whereas a positive WCB value indicates that more left-wing-clipped flies were eaten compared to right-wing clipped flies (figure 1F, see Methods). The mean WCB values for control experiments with all fly genotypes used in this study were not significantly different from zero, indicating that neither right- nor left-wing clipping biased survival chances in this assay (figure 1G, electronic supplementary material, figure S1C).

During prey survival competition trials between different fly genotypes, the side of wing clipping (left or right) was alternated between GF-silenced flies and GF driver-only controls to further minimize bias. For each fly genotype match-up, a prey consumption index (PCI) value was calculated for each trial (see Methods). A positive PCI indicates that more GF-silenced flies were eaten compared to control flies, with a larger absolute value corresponding to a larger difference between the number of GF-silenced and control flies eaten (figure 1F). Both GF1>+ (control) versus GF1>Kir2.1 (GF-silenced) and GF2>+ versus GF2>Kir2.1 fly genotype match-ups yielded significantly positive mean PCI values, indicating that more GF-silenced flies were consumed relative to control flies (figure 1G). GF1>Kir2.1; Gal80ts flies were also preferentially eaten compared to control flies, though with a slightly smaller mean PCI (figure 1G). These results establish a direct link between GF activity and fly survival under real predation conditions.

### (b) Giant fibre silencing increases the likelihood of fly capture

**Figure 2.**
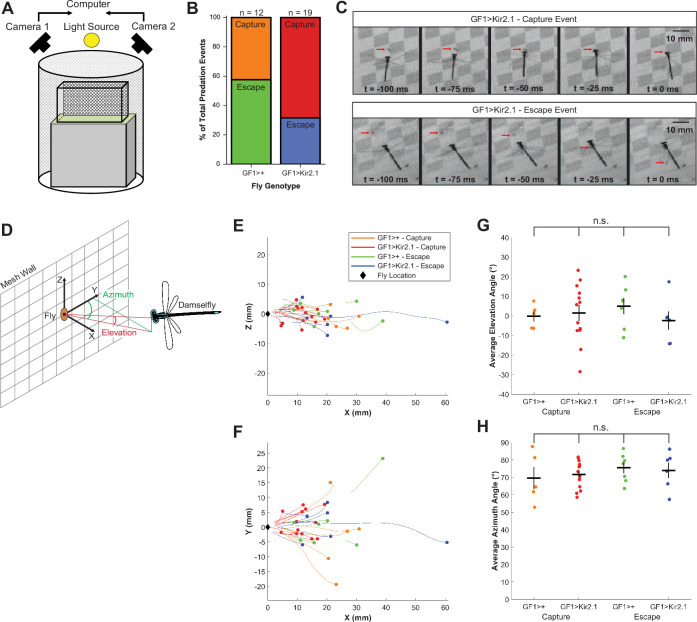
GF silencing increases the likelihood of fly capture. (A) Set-up of population assay for high-speed videography of predation events. Predation events were analysed from zoomed-in views of the entire population cage depicted in figure 1E. (B) Proportion of recorded predation events that resulted in fly escape or capture outcomes for GF driver-only control and GF-silenced flies. GF1>+ (*n* = 12), GF1>Kir2.1 (*n* = 19); *n* = number of attack events. (C) Representative video frames showing GF1>Kir2.1 fly capture and escape events. (D) Reference frame coordinates for tracked damselfly attacks. (E,F) Damselfly attack trajectories in the XZ (E) and XY (F) planes. Black diamonds represent fly initial positions. Coloured dots and lines represent the damselfly initial position and trajectories for different combinations of fly genotypes and escape outcomes. (G,H) Average damselfly elevation (G) and azimuth (H) angles for each predation attempt. Coloured dots represent individual predation events, black crosses represent mean ± s.e.m; n.s. = not significant, one-way ANOVA followed by Tukey HSD *post hoc* test.

Having established that GF-silenced flies are eaten more frequently than driver-only control flies under naturalistic conditions, we next examined *how* the GFs contribute to prey survival. To determine whether the greater consumption of GF-silenced flies relative to control flies was directly due to deficiencies in fly escape performance, we video recorded damselfly predation events in a behaviour chamber over an 8 h period (figure 2A). As fly wing-clipped side, and hence discrimination between different genotypes, was difficult to determine from the video recordings, the chamber was populated by 200 flies (100 left- and 100 right-wing clipped) of a single genotype during each experiment. Comparing experiments with different genotypes, we found that the percentage of successful escape outcomes for GF-silenced flies was only half of that for GF driver-only control flies on an individual encounter basis, which corroborates our findings from the prey survival competition assays that the GFs promote fly survival during naturalistic predator attacks (figure 2B).

We observed that during an aerial attack sequence, damselflies hover at around the same height as a fly perching on the mesh wall before darting forward to grab the fly with their legs (figure 2C). We considered the period after the hover when the damselfly is moving toward the targeted fly as the ‘attack’ and we quantified damselfly attack trajectories by calculating the elevation and azimuth angles of the damselfly relative to the targeted fly during this period (figure 2D). We found no statistically significant differences in average elevation or azimuth angles across the duration of the attack sequence between prey genotypes (either GF driver-only control or GF-silenced) or capture/escape outcomes (figure 2E,F,G,H, electronic supplementary material, figure S2Aand S2B). This implies that differences in damselfly attack trajectories are not accountable for the poorer escape performance of GF-silenced flies compared to GF driver-only control flies.

### (c) Giant fibre-silenced flies are captured by slower attacks and allow closer damselfly approach

**Figure 3.**
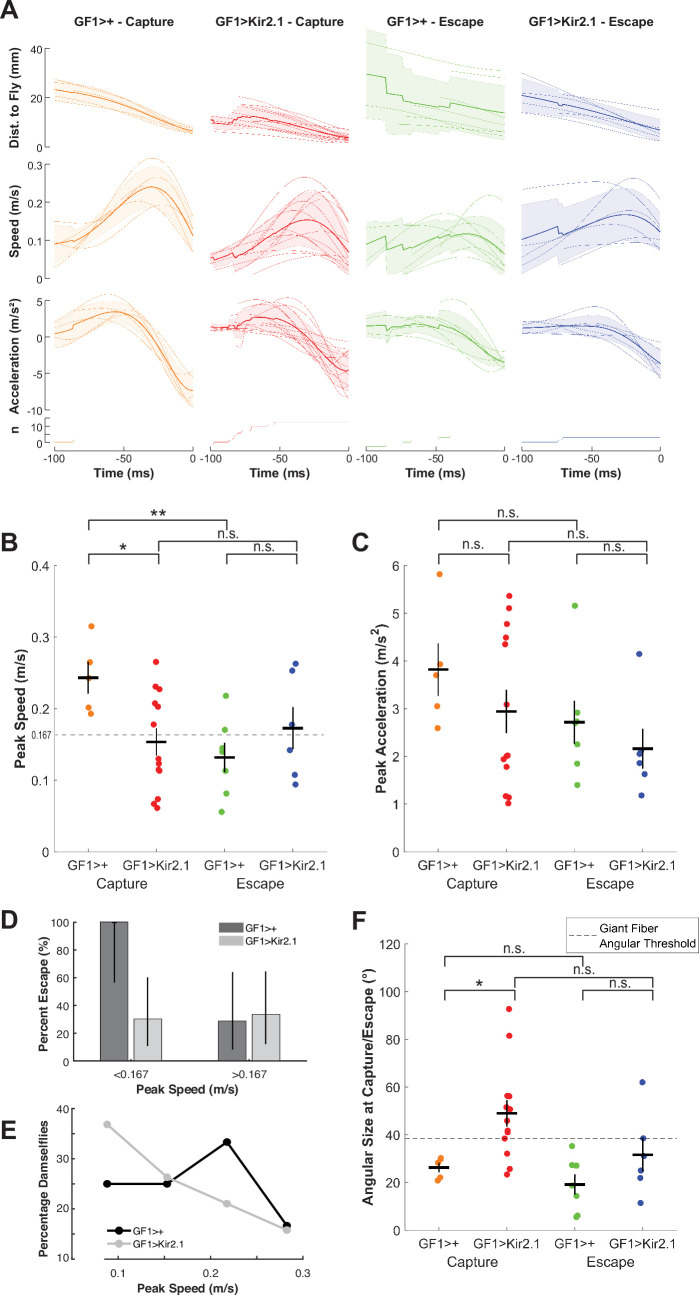
GF-silenced flies are captured by slower attacks and allow closer damselfly approach. (A) Kinematics of damselfly attack trajectories for predation events involving GF-silenced (red, blue) and control (orange, green) flies, clustered by attack outcome (fly capture or escape). Time = 0 represents the time of either contact or escape. Thin lines represent individual attack events, and thick lines represent average values. Shaded regions represent the standard deviation. Number of predation events contributing to each time point is indicated in the bottom row; note that some averages have step changes due to averaging trajectories of different durations. (B) Damselfly peak speeds during attacks. Coloured dots represent individual predation events, black crosses represent mean ± s.e.m. GF1>+ Capture vs. GF1>Kir2.1 Capture (*p = 0.0176), GF1>+ Capture vs. GF1>+ Escape (**p = 0.0047), GF1>Kir2.1 Capture vs. GF1>Kir2.1 Escape (*p* = 0.5788, n.s.), GF1>+ Escape vs GF1>Kir2.1 Escape (*p* = 0.2677, n.s.); *p*-values from two-sample *t*‐test. (C) Damselfly peak accelerations during attacks. Coloured dots represent individual predation events, black crosses represent mean ± s.e.m. GF1>+ Capture vs GF1>Kir2.1 Capture (*p* = 0.2989, n.s.), GF1>+ Capture vs. GF1>+ Escape (*p* = 0.1525, n.s.), GF1>Kir2.1 Capture vs. GF1>Kir2.1 Escape (*p* = 0.3057, n.s.), GF1>+ Escape vs. GF1>Kir2.1 Escape (*p* = 0.3987, n.s.); *p*-values from two-sample *t*‐test. (D) Percentage of GF1>+and GF1>Kir2.1 flies that escaped damselfly attacks with peak speeds slower or faster than the mean attack speed for all trials across all four conditions (0.167 m s^–1^, dashed line in figure 3B). Note the large reduction in GF>Kir2.1 flies that were able to escape the slower attack speeds (<0.167 m s^–1^) that all control flies were able to escape. Error bars represent 95% confidence intervals calculated with the Wilson score interval. (E) Distribution of peak attack speeds reached by damselflies during GF1>+and GF1>Kir2.1 predation events. (F) Angular size of damselfly on fly’s retina at time of contact or escape. Coloured dots represent individual predation events, black crosses represent mean ± s.e.m. GF1>+ Capture vs. GF1>Kir2.1 Capture (*p = 0.0249), GF1>+ Capture vs. GF1>+ Escape (*p* = 0.2197, n.s.), GF1>Kir2.1 Capture vs. GF1>Kir2.1 Escape (*p* = 0.0858, n.s.), GF1>+ Escape vs. GF1>Kir2.1 Escape (*p* = 0.1488, n.s.); p-values from two-sample *t*‐test. Dashed line indicates the mean angular size at which looming stimuli were previously determined to drive a GF spike and take-off [18].

We next analysed damselfly attack kinematics from the recorded predation events to extract visual features of aerial predator attacks that the GFs are responsive towards [26–28]. In assays using either GF driver-only control or GF-silenced flies, fly escape outcomes corresponded to damselfly attacks that were approximately constant in approach velocity (figure 3A). For attacks resulting in control fly capture, however, damselflies accelerated as they approached their target prey, with speeds peaking at 30 ± 7 ms before time of contact (figure 3A). A closer look at individual predation events revealed a bimodal distribution in peak speeds and accelerations for GF-silenced capture events (figure 3B,C). While damselfly peak speeds corresponding to control fly capture outcomes were significantly higher than those for escape outcomes, this difference was abolished for GF-silenced flies (figure 3B). Similarly, damselfly peak acceleration values for control capture outcomes were generally higher than for escape outcomes, while values varied considerably for GF-silenced capture outcomes (figure 3C). Thus, compared to control flies, GF-silenced flies exhibit a reduced ability to escape for slower damselfly attack peak speeds and accelerations. In other words, GF-silenced flies fail to escape in time when confronted with attack speeds at which they would normally be able to react (figure 3D, electronic supplementary material, figure S3A). Interestingly, possibly as an adaptive response to fly prey escape impairment, damselflies in the GF-silenced trial tended to attack at slower speeds compared to the trial with GF driver-only control flies (figure 3E).

We observed that damselflies were able to approach more closely to GF-silenced flies, especially for the trials that ended with capture (electronic supplementary material, figure S3B). It was previously shown that a GF spike, and consequently a short mode escape take-off, is triggered once a looming stimulus reaches an angular size threshold of approximately 40° on the fly’s retina [18]. We examined the relationship between the maximum angular size of a damselfly subtended at its closest distance to the fly and fly escape outcomes for both genotypes. To do this, we used the damselfly head width (electronic supplementary material, figure S3C), the widest part of the damselfly body from the fly’s perspective during a direct attack, to convert the distance of the damselfly at the point of fly capture or escape to an estimated angular size that the damselfly would subtend on the fly’s retina (figure 3F). Damselflies for GF-silenced fly capture outcomes were the only ones that consistently and considerably surpassed the GF activation threshold (figure 3F). This observation suggests that a role of the GF is to ensure that the fly escapes before a predator comes close enough to capture it with high probability, and that this is a unique role for this single pair of giant axon descending neurons—there is no other fail-safe or redundant circuit for this function.

### (d) Giant fibre increases escape likelihood by promoting the shorter-duration escape take-off sequence

**Figure 4.**
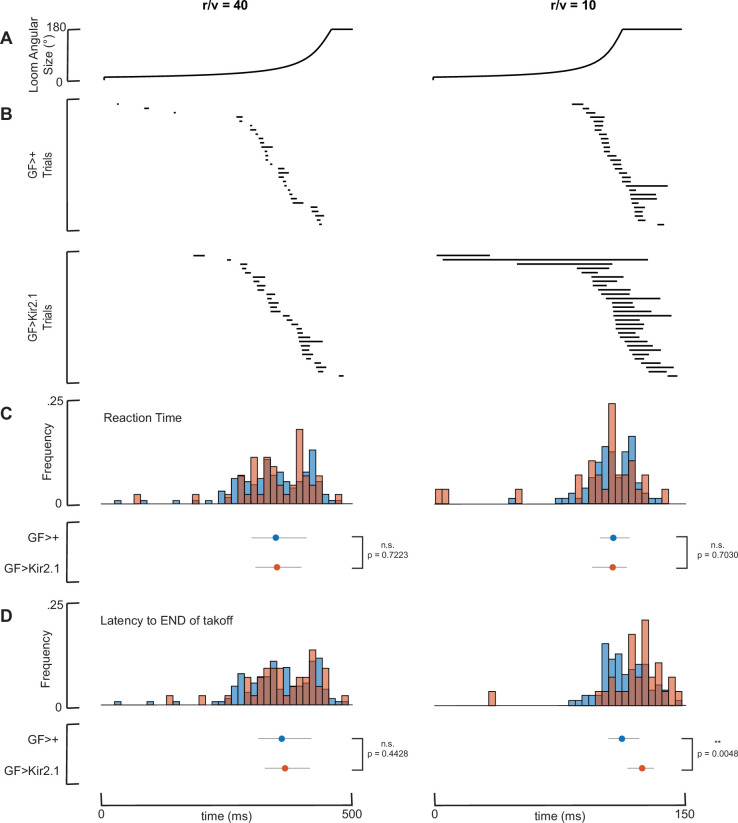
GF increases escape likelihood by promoting the shorter-duration escape take-off sequence. (A–D) GF>+and GF>Kir2.1 fly escape sequences for *r/v* = 40 (left column) and *r/v* = 10 (right column) loom expansion speeds in FlyPEZ assays. Note the shorter time axis range for the *r/v* = 10 column. (A) Time course of looming stimulus angular size expansion. (B) Take-off sequence ethograms. Dark lines mark the duration of the take-off sequence, from wing lift initiation to loss of ground contact for representative individual flies, *n* = 29 flies per genotype per loom expansion speed. (C) Histogram (above) and median ± interquartile range (below) of reaction time (latency to start of take-off sequence). Data are pooled from experiments with both GF1 and GF2 drivers. GF>+, *r/v* = 40 (*n* = 141), GF>Kir2.1, *r/v* = 40 (*n* = 48), GF>+, *r/v* = 10 (*n* = 80), GF>Kir2.1, *r/v* = 10 (*n* = 29); n.s. = no significance, two-sample *t*‐test. (D) Histogram (above) and median ± interquartile range (below) of latency to the *end* of the take-off sequence. Data are pooled from experiments with both GF1 and GF2 drivers. GF>+, *r/v* = 40 (*n* = 141), GF>Kir2.1, *r/v* = 40 (*n* = 48), GF>+, *r/v* = 10 (*n* = 80), GF>Kir2.1, *r/v* = 10 (*n* = 29); n.s. = no significance, **p < 0.01, two-sample *t*‐test.

The GFs triggering escape at a certain size threshold as a way to prevent predators from getting too close is at odds with the idea that giant axons facilitate fast transmission, enabling the animal to reduce their reaction time (i.e. time from the start of the stimulus to the start of the behavioural response). To directly assess whether fly reaction times were altered by GF silencing, we re-analysed the behavioural responses of GF driver-only control and GF-silenced flies that were challenged with virtual looming stimuli in the FlyPEZ (figure 4A). We manually annotated the frame in which the escape take-off sequence started (when the fly’s wing first begins to elevate) and when the sequence concluded (when the first tarsus of the two middle legs leaves the ground) (figure 4B). We consider the reaction time to be the former (earlier) of these two time points, and the escape time to be the latter (later) of these two time points. We found that the range of reaction times was quite large and that there was no significant difference in mean reaction time between GF-silenced and GF driver-only control flies for either the slow (*r/v* = 40) or fast (*r/v* = 10) stimulus (figure 4C). When we examined the timing of the end of the escape sequence when the fly lifts off, however, we found that the mean escape time was significantly shorter for control flies compared to GF-silenced flies in the case of the faster-looming stimuli (*r/v* = 10) (figure 4D). This implies that the fitness consequence of our earlier observation that GF-driven escape sequences are shorter than non-GF-mediated ones (figure 1C) is that even without reacting sooner, flies can still escape quicker, and that this is likely relevant to surviving naturalistic damselfly attacks. Taken together, the role of the *Drosophila* GFs is not to reduce reaction time during a predator attack but rather to mediate action selection, ensuring that the fly elects to use its short mode take-off sequence to get off the ground in time if and when a looming threat gets close enough that capture is imminent.

## 3. Discussion

Over the course of evolution, predation risk in the wild exerts large selection pressures on the form and function of prey escape circuits. A hallmark of escape circuit architecture is degeneracy—in crayfish, teleost fish, and flies, both giant and non-giant neuron-mediated pathways connect sensory detection of a predator to motor neurons coordinating an escape [14,18,29], and this architecture is preserved, even in fly strains that have been lab-reared without predators for several generations. The ability to recruit multiple parallel pathways in different combinatorial patterns lends flexibility to rapid escape responses in each predation scenario. However, the metabolic cost of maintaining a neuron increases proportionally to the neuron’s volume [2–4]. As such, including a giant neuron in a degenerate network is likely to be selected against unless its presence somehow increases the animal’s fitness beyond the contributions of non-giant-mediated circuits.

Despite the ubiquity of predator–prey interactions in ecological networks, few attempts have been made to determine the survival value and role of giant interneurons that mediate predator evasion reponses under ecologically relevant conditions. Two decades ago, giant-independent crayfish tail flips were found to be less effective than giant-mediated tail flips at preventing capture during dragonfly nymph attacks [30]. A more recent study in zebrafish also demonstrated the life-preserving role of the giant Mauthner cell when subjected to damselfly nymph predation [31]. Both studies demonstrated that the role of the giant neurons in these systems was to reduce the time between predator detection and the first escape action, improving the prey’s likelihood of escaping before capture. This behavioural role for giant neurons matches their biophysical adaptation of enlarged axon diameter, which increases action potential speed by decreasing the axial resistance of the main neuron cable [32]. It has thus been presumed that the role of giant neurons in escape circuits is to reduce prey reaction time.

Our results challenge this narrative as the sole explanation for giant interneurons in escape circuits. Previous studies have shown that the anatomy of the *Drosophila* GFs and their outputs are organized to drive an extremely fast take-off action. GF axons are approximately 7 μm in diameter [33], which is considerably larger than any other profile in the neck connective [34]. In the fly’s ventral nerve cord, the GFs are electrically coupled with the motor neuron (tergotrochanteral motor neuron, TTMn) that activates the mesothoracic leg jump muscles as well as with an interneuron (peripheral synapsing interneuron, PSI) that projects cholinergic chemical synapses onto the large wing depressor muscles (dorsolateral muscles, DLMs) [35]. A single action potential in the GF drives a muscle potential in the TTM in under a millisecond and in the DLMs less than half a millisecond later [34]. The conduction time along the GF itself has been measured at 0.29 ms [33]. GF activation thus rapidly activates a stereotyped muscle activity pattern driving the fly to jump, then depress its wings and initiate flapping flight. However, we find that GF silencing does not alter reaction time, measured as the latency to when the take-off sequence *starts*. Instead, the GF enforces the selection of this stereotyped shorter-duration escape take-off sequence, reducing the latency to when the take-off sequence *ends*. In *Drosophila*, the fast transmission speed facilitated by the large diameter of the GF axon performs an action selection role that short circuits parallel pathways that would drive longer escape take-off sequences, enforcing use of the shorter, life-saving action. Our findings are consistent with theoretical and anatomical analyses that larger axons promote higher information rates, reducing the need to spread a signal over multiple low-rate channels whose messages would take time to integrate downstream [36].

Previous circuit-level analysis revealed that the GF integrates input from two different visual projection neuron types, LPLC2 and LC4 [26–28]. These neurons provide information about the angular size and expansion velocity of looming stimuli, respectively, to trigger a short mode escape when a rapidly expanding virtual looming stimulus reaches about 40° on the fly’s eye [27,28]. Our findings also support this circuit model at a behavioural ecology level. We show that the GFs are necessary for flies to consistently escape before an attacking damselfly’s head reaches a size of 40° on its retina. This critical size of 40° corresponds to when the damselfly is about 3 mm away from the fly, which is within the range at which the damselfly can comfortably extend its four front legs to grab the fly. We note that in our observed predator–prey interactions with GF-silenced flies, the fly was captured in all but one of the events in which the damselfly approached GF-silenced flies more closely than this GF angular threshold. However, the cosmopolitan *D. melanogaster* also inhabits niches, including urban indoor environments, that are outside the damselflies’ ecological range near freshwater systems. As such, fly escape responses are likely adapted to attack parameters that are common among a broader range of predators as opposed to damselfly attack features *per se*.

Our working model of the GF and its companion smaller-axon take-off-inducing descending neurons (DNs) is that the GF’s looming-size activation threshold is higher than that of the smaller-axon DNs [18]. In this model, slower looming activates just the smaller-axon pathways, eliciting the non-GF long mode take-off. On the other hand, faster looming activates both the smaller-axon pathways and the GF, with the smaller-axon DNs activated marginally earlier at the lower threshold. To ensure a short mode take-off, the GF signal thus needs to arrive at the take-off-orchestrating motor neurons earlier than the smaller-axon signal. Our previous model and findings here collectively suggest that this is accomplished in two ways: one is the axon gigantism that allows for faster conduction along the GF, and the second is a longer ‘motor delay’ in the smaller-axon pathway, which connectome data indicate could be due to the smaller-axon DNs contacting VNC interneurons, rather than motor neurons directly as the GF does [37]. The temporal competition can thus be viewed as not between the reaction times of prey and predator (see figure 4) but rather between the conduction speeds along the GF- and smaller-axon DN-mediated pathways.

By combining approaches in behavioural neuroscience and ecology, we provide experimental evidence that the GF promotes prey fitness by enhancing fly escape performance when challenged with damselfly attacks in a naturalistic setting. Our analysis of damselfly attack kinematics and fly escape timing also corroborates, in a naturalistic setting, our previous findings from experiments with virtual looming stimuli that the GF drives timely escape responses by integrating multiple looming parameters, including the angular size of the predator on the fly’s retina [18]. We conclude that despite the presence of parallel escape pathways, GF-mediated fast escapes are still strongly accountable for successful predator evasion and survival under ecologically relevant conditions.

## 4. Material and methods

### (a) *Drosophila melanogaster* strains

We used 2- to 5-day-old female *D. melanogaster* reared on standard cornmeal fly food at 22–25**°**C and 50% humidity with a 16 h light/8 h dark cycle. The fly stocks were used in this study are listed in table 1.

**Table 1.** Fly stocks.

genotype	source
*DL* – wild-type from Pasadena, California, USA	M.H. Dickinson, Caltech
*GF_1-split-GAL4* (*R17A04_p65ADZp; R68A06_ZpGdbd*)	[18]
*GF_2-split-GAL4* (*R14A01_p65ADZp; R79H02_ZpGdbd*)	[27]
w+;; pJFRC49−10XUAS-IVS-eGFPKir2.1	[18]
*w+; UAS-Kir2.1; tubP-Gal80ts*	Bloomington *Drosophila* Stock Centre no. 6596 and no. 7017

The *tubP-Gal80ts* transgene is not functional when crossed with split-GAL4 driver lines such as GF1 and GF2 [38]. The *pJFRC49−10XUAS-IVS-eGFPKir2.1* construct has a higher UAS copy number compared to the *UAS-Kir2.1; tubP-Gal80ts* construct and should produce a stronger neuronal silencing effect.

### (b) FlyPEZ assay

We used a high-throughput behavioural assay, FlyPEZ, to record the responses of unrestrained flies presented with computer-generated looming stimuli that had the same expansion kinematics as a virtual object of radius, *r*, approaching the fly with a constant velocity, *v* [24]. In accordance with a previous study [39], the time course of the size of the looming stimulus can be denoted by the size-to-speed ratio, *r/v*, of the virtual object. That is, a large object approaching the fly quickly would have the same expansion rate on the fly’s eye as a small object approaching slowly, if they start with the same angular size and have the same *r/v* value. In the FlyPEZ, individual flies were released through an automated gate onto a prism where they were presented with a dark disk stimulus projected onto a white dome. The dark disk expanded to a fixed size at different looming rates characterized by their *r/v* ratio (fast looming, *r/v* = 10 ms; slow looming, *r/v* = 40 ms). If flies failed to take off upon stimulus presentation, the stage was cleared before the release of the subsequent fly. All experiments were conducted during the 4 h time window before incubator lights were switched off, which coincides with the flies’ evening activity peak. A single stimulus was presented per fly, and videos were recorded at 6000 frames per second under 740 nm infrared illumination. To quantify the duration of the escape take-off motor sequence, videos were manually annotated for the start (first frame of wing raising) and end (first frame when the two middle ‘jumping’ legs left the ground) of the escape sequence. A total of 1474 flies were screened. For figure 1C, to minimize bias towards *r/v* = 10 or *r/v* = 40 data after pooling, the *n* in the larger group was decreased by randomly selecting points to remove in order to match the n in the smaller group.

### (c) Prey survival competition assays

The day before prey survival competition and predation high-speed videography (below) experiments, flies were cold anaesthetized for a maximum of 20 min while either their left or right-wing tips were excised using dissection scissors. Male and female damselflies belonging to the genus *Ischnura* were wild-caught from a pond within the Janelia Research Campus in Ashburn, Virginia, USA, using a butterfly net. Damselfly species were likely *Ischnura posita* (fragile forktail) and/or *Ischnura elegans* (blue-tailed damselfly) based on the pattern of body coloration and markings. Damselflies were kept in 100 mm plastic Petri dishes on ice for 1−2 h before experiments. All experiments were conducted between the months of July and September.

#### (i) Experimental design

For prey survival competition assays, we released 100 GF >+ (GF split-GAL4 driver lines crossed to DL) flies, 100 GF >Kir2.1 or GF >Kir2.1; Gal80ts (GF split-GAL4 driver lines crossed to either Kir2.1 or Kir2.1; Gal80ts) flies, and 10 damselflies into a 15 × 15 × 20 cm clear plastic behaviour chamber. For WCB control assays, we released 100 left-wing clipped and 100 right-wing clipped flies of the same genotype into the chamber with 10 damselflies. Each behaviour chamber had a mesh wall on the long face to facilitate fly perching and the equilibration of internal and external conditions. A wet paper towel was taped down to the floor of each chamber at the start of each assay period to minimize animal dehydration.

#### (ii) Experimental conditions

To simulate naturalistic conditions, we placed the boxes inside an environmental room that was illuminated to outdoor conditions and wallpapered with naturalistic scenery. Temperatures in the room cycled between 31**°**C during the day and 19**°**C overnight. Four behaviour chambers were assayed simultaneously during each 8 h assay period, which began at around 10.00 and ended at around 18.00.

#### (iii) Fly collection and counting

At the end of each experimental period, flies were anaesthetized using FlyNap (a mixture of 50% triethylamine, 25% fragrance, 22.63% ethanol, 1.25% 2-propanol and 1.13% methanol purchased from the Carolina Biological Supply Company). Anaesthetized flies from each chamber were then collected in a flask connected to a vacuum line and left in a 4**°**C refrigerator for a few hours before counting. The WCB index and PCI values were calculated using the following formulas:


$$\begin{array}{rl} WCB & =\frac{No.\;\;of\;left\;wing\;clipped\;flies\;eaten\;–\;No.\;\;of\;right\;wing\;clipped\;flies\;eaten}{No.\;\;of\;left\;wing\;clipped\;flies\;eaten\;+\;No.\;\;of\;right\;wing\;clipped\;flies\;eaten}\\ PCI & =\frac{No.\;\;of\;GF-silenced\;flies\;eaten\;–\;No.\;\;of\;GF-wt\;flies\;eaten}{No.\;\;of\;GF-silenced\;flies\;eaten+No.\;\;of\;GF-wt\;flies\;eaten} \end{array}$$

### (d) High-speed videography of damselfly predation events

Similar to the WCB assays, a behavior chamber containing 100 left wing-clipped and 100 right-wing clipped flies of the same genotype was released into a chamber with 10 damselflies. The chamber was placed on a podium covered with artificial green turf within a cylindrical enclosure lined with checkered wallpaper to provide optic flow. Two high-speed cameras (Photron SA−1) were positioned orthogonal to each other around the enclosure, with both cameras focused on the chamber’s mesh wall. Assays were performed outside the indoor environmental arena at room temperature to facilitate video recording. Throughout an 8 h assay period, an experimenter observed the cameras’ live feeds on a PC monitor and manually triggered the cameras to record predation events. Videos were recorded at 1000 frames per second using Photron software. GF-wild-type and GF-silenced flies were assayed separately on consecutive days. After video acquisition, videos were screened for true predation attempts using the following criteria: (i) the damselfly hovers in place behind the perching fly during attack sequence initiation, (ii) moves forward towards target, and (iii) forms a basket with its legs to grab the fly. If any one of these three criteria was not met, the video was discarded. An attack event was scored as a capture if the damselfly grabbed the fly off the mesh wall. An attack event was scored as an escape if the fly jumped away from the wall before the damselfly could reach it. Flies that were initially caught but eventually freed themselves from the damselflies’ grasp were still scored as being captured.

### (e) Predation video digitization and three-dimensional kinematic analysis

Damselfly head, damselfly tail, and fly body centroid points were marked in each video frame using DLTdv software [40]. Damselfly attack trajectories were analysed using custom scripts in MATLAB. Each component axis was passed through a Butterworth filter followed by a Savitzky–Golay filter, and attack kinematics parameters were calculated. Due to left–right symmetry, the absolute values of the azimuthal plane coordinates (XY) were used to calculate average trajectory azimuth angle, constraining the angle between 0° and 180°. The angular size of the damselfly on the fly’s retina at the time of escape or capture (θ) was calculated using the formula θ = 2tan^-1^(*r*/*d*) where 2 *r* is the damselfly head width and *d* is the Euclidean distance between the damselfly and the fly at the time of escape or capture [18].

### (f) Quantification and statistical analysis

For prey survival competition assays (figure 1, electronic supplementary material, figure S1), Student’s one-sample *t*‐test was used to determine statistically significant differences from zero for PCI and WCB index scores. For damselfly attack elevation and azimuth analysis (figure 2), one-way ANOVA followed by Tukey HSD *post hoc* test was used to determine statistically significant differences between all groups. For damselfly attack kinematics analysis (figure 3, electronic supplementary material, figure S3), Student’s two-sample *t*‐test was used to determine statistically significant differences between groups of interest. For FlyPEZ assays (figure 4), Student’s two-sample *t*‐test was used to determine statistically significant differences between fly genotypes.

## Data Availability

Data from this study as well as custom MATLAB code used to plot the figures are available at [41]. Supplementary material is available online [42].
